# Haplotype-resolved assembly of a pig genome using single-sperm sequencing

**DOI:** 10.1038/s42003-024-06397-x

**Published:** 2024-06-18

**Authors:** Yongchao Niu, Xinhao Fan, Yalan Yang, Jiang Li, Jinmin Lian, Liu Wang, Yongjin Zhang, Yijie Tang, Zhonglin Tang

**Affiliations:** 1 Kunpeng Institute of Modern Agriculture at Foshan, Agricultural Genomics Institute, Chinese Academy of Agricultural Sciences, Foshan, China; 2 Shenzhen Branch, Guangdong Laboratory for Lingnan Modern Agriculture, Agriculture Genomics Institute at Shenzhen, Chinese Academy of Agricultural Sciences, Shenzhen, China; 3 GuangXi Engineering Centre for Resource Development of Bama Xiang Pig, Bama, China; 4 Key Laboratory of Livestock and Poultry Multi-Omics of MARA, Agricultural Genomics Institute at Shenzhen, Chinese Academy of Agricultural Sciences, Shenzhen, China; 5 Biozeron Shenzhen, Inc., Shenzhen, China

**Keywords:** Genomics, Genome

## Abstract

Single gamete cell sequencing together with long-read sequencing can reliably produce chromosome-level phased genomes. In this study, we employed PacBio HiFi and Hi-C sequencing on a male Landrace pig, coupled with single-sperm sequencing of its 102 sperm cells. A haplotype assembly method was developed based on long-read sequencing and sperm-phased markers. The chromosome-level phased assembly showed higher phasing accuracy than methods that rely only on HiFi reads. The use of single-sperm sequencing data enabled the construction of a genetic map, successfully mapping the sperm motility trait to a specific region on chromosome 1 (105.40–110.70 Mb). Furthermore, with the assistance of Y chromosome-bearing sperm data, 26.16 Mb Y chromosome sequences were assembled. We report a reliable approach for assembling chromosome-level phased genomes and reveal the potential of sperm population in basic biology research and sperm phenotype research.

## Abstract (web summary)

An improved pig genome assembly is reported, using single sperm sequencing to reconstruct chromosome haplotypes, and with improved Y chromosome sequence.

## Introduction

The dissimilarity between homologous chromosomes within a diploid genome influences various aspects of genetic analysis, including genome annotation, allele expression, and the exploration of inter-individual homologous chromosome variation^1–3^. Single-cell gamete sequencing has the potential to enable chromosome-scale phased genome assembly and personalized genetic map construction, thereby providing valuable insights into personalized genetics^4,5^. Due to technological limitations, the fusion of diploid genomes into a pseudo-haploid sequence has been a common practice for an extended period, inadvertently creating challenges for subsequent research endeavors. With the advancement of sequencing techniques, particularly the progress made in long-read sequencing technologies such as Pacific Biosciences (PacBio) and Oxford Nanopore sequencing, the possibility for haplotype assembly using long reads has become apparent. Among the available options for phase assembly software, Falcon-unzip^6^, and Supernova^TM^ assembler^7^ (10x Genomics) make use of the overlap of long reads to determine haplotype phases. However, these methods are restricted to resolving haplotype differences over short distances. Although Falcon-phase can generate longer haplotypes by incorporating Hi-C data, it falls short of achieving chromosome-level phased assembly^8,9^. Hifiasm can identify parental-specific read bins, enabling the assembly of diploid genomes effectively^10^. Dipasm utilizes HiFi and Hi-C data to achieve chromosome-level phased assemblies^11^. However, significant challenges still exist in the pursuit of comprehensive chromosome-level phased genomes, especially in cases where a chromosome contains numerous regions of low heterozygosity. A method that combines stranded short-read RNA-seq with long-read sequencing has demonstrated effectiveness in phasing the human genome^12^. However, the broader application of this approach has been hindered by the challenge of obtaining strand-specific sequencing data. Fu et al. have developed a methylation-based haplotype phasing method called MethPhaser which they used to phase a human genome^13^. Shi et al. utilized 12 pollen samples to assemble a pear genome^14^, while Kirkness et al. used 96 sperm cells to phase the HuRef human genome^15^, thereby demonstrating the feasibility of gamete-based genome assembly. However, the current limitations of sequencing technologies continue to impede the attainment of high-quality assemblies. Trio binning, which relies on Illumina short reads from both parental genomes to differentiate haplotypes within offspring’s long reads, is utilized to construct complete diploid assemblies^1^. However, in the case of mammals, the accessibility of parental genetic information is frequently constrained, thereby limiting the applicability of trio binning.

The first reference pig genome of a female Duroc pig was published in 2012^16^, and subsequently, several other pig genomes have been reported^9,17–21^. However, the absence of chromosome-level phased genomes in pigs persists due to technological limitations. In this study, we sequenced a male Landrace pig using PacBio HiFi and Hi-C technologies and 102 of its sperm with single-cell sequencing technology. A phased genome at the chromosomal level was obtained using phased markers and phased long reads. The assembly exhibited high accuracy in phasing. Additionally, using the sperm data, a genetic map was constructed and then used for studying the sperm motility trait. Moreover, the insights gained from Y chromosome-bearing sperm data proved instrumental in advancing pig Y chromosome assembly. Overall, our research revealed the importance of single sperm sequencing in phased genome assembly and sperm phenotype research.

## Results and Discussion

### The strategy for obtaining complete haplotypes of sperm

**Fig. 1:**
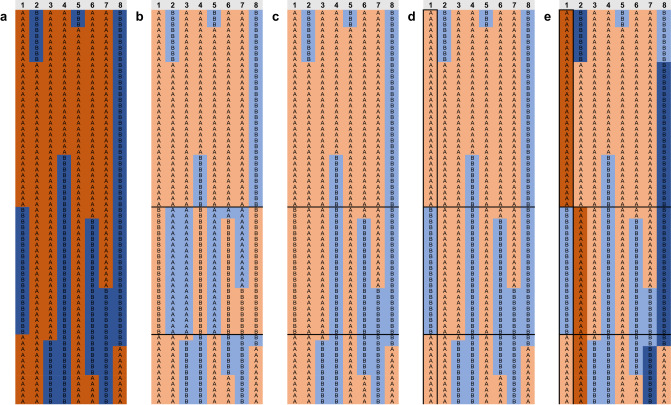
Schematic diagram of sperm haplotype inference. Numbers 1-8 represent the chromosomes of No.1-8 sperms, and the parental genotype was indicated by A, B, and orange and blue colors, respectively. The black horizontal lines represent false recombination sites in the population. **a** The real chromosome recombination map of the eight sperm. **b** The recombination map after genotyping using sperm No.1 as the reference. The same genotype with No.1 sperm was marked as orange, and the different genotype was marked as blue. False recombination occurs at the horizontal line with all sperm (except No. 1) recombined at this position, which was caused by the real recombination of No. 1 sperm. **c** The recombination map of No. 2-8 sperm after the false recombination site correction. **d** The recombination map after the correction of all sperm. **e** The complete haplotype path is based on the population genotype information, which was indicated in dark orange and dark blue.

In the mammalian meiosis process, after two rounds of cell division following DNA replication, homologous chromosome pairs exchange some genetic materials, resulting in two haploid cells and then generate four genetically unique gametes (sperm or egg) after the sister chromatids segregate^4^. Sperm has a parental genotype chimeric composition, as shown in Fig. 1a and Supplementary Fig. 1. There are several methods to obtain haplotypes for sperm data; for example, Carioscia et al. developed the rhapsodi method suitable for low coverage of single-gamete sequence analysis^22^. Li et al. developed Hapi, which utilizes sperm data to obtain haplotypes by employing the PHMM (pairwise Hidden Markov Model) method^23^. Lyu et al. developed sgcocaller software, which outperforms the Hapi algorithm in accuracy and performance, providing great efficiency for sperm research^24^. In this study, we sequenced 102 sperm cells with an average depth of 10.05X and sequenced the blood samples from the donor boar with 95.12X, which allowed us to directly infer sperm haplotypes, as shown in Fig. 1. For example, in Fig. 1b, we can phase the genotype of sperm No. 2–8 by comparing their genotypes with sperm No. 1 (same genotype with same color). Based on the recombination information of sperm 2–8, we can easily identify false recombination sites, as shown by the black horizontal line in Fig. 1. These false recombination sites are characterized by recombination occurring in sperm 2–8 (Fig. 1b). As recombination occurs randomly in the population, these abnormal recombination sites are easily identified, which is actually caused by the true recombination of sperm No. 1, leading to false recombination of all other sperm cells at that location. By correcting these false recombination sites, we can obtain real recombination sites in the population (Fig. 1c, d), and then obtain chromosome-level haplotype markers for subsequent phased genome assembly (Fig. 1e).

### Initial genome assembly and acquisition of haplotype markers

**Fig. 2:**
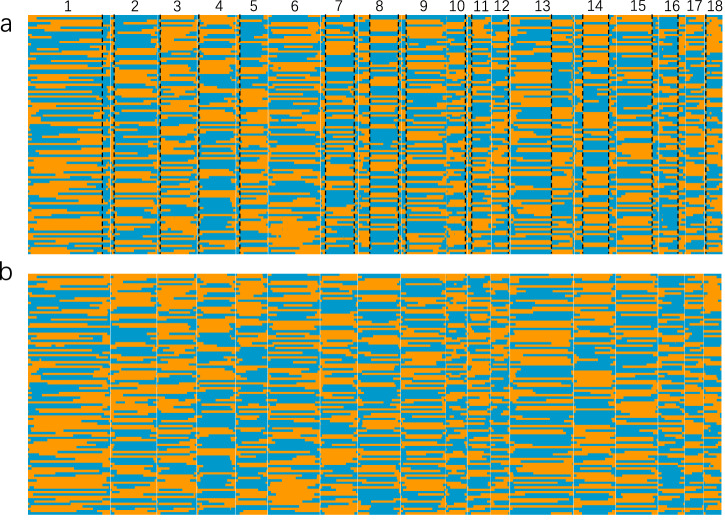
Inferring parental haplotype using sperm population. **a** The preliminary recombination map inferred by using sperm S18-162 as the reference, the chromosomes are divided by the white vertical lines, and the vertical black dotted lines represent the potential false recombination site. **b** The final recombination map after correcting the false recombination sites and filtering suspicious recombination.

For the initial genome assembly, 237.79 Gb (95.12X) Illumina 350 bp insertion library data, 89.94 Gb (35.98X) Hi-C data, and 94.26 Gb (37.70X) Pacbio HiFi data (Supplementary Table 1) were obtained from the blood of the Landrace boar. We employed the Falcon unzip pipeline, yielding an initial assembly with a contig N50 of 29.52 Mb and a length of 2.58 Gb (Supplementary Table 2). Firstly, the Illumina 350 bp insertion library data from the blood sample of the same pig that was aligned to the initial genome assembly and 5.02 million heterozygotes sites across the autosomes were detected. The density distribution of heterozygous sites displayed uneven patterns across chromosomes (Supplementary Fig. 2). Certain regions of chromosomes exhibited lower densities of heterozygous sites when compared with other chromosome regions, reflecting the inbreeding process of Landrace pigs leading to some regions of the sequenced genome becoming homozygous. Subsequently, the initial assembly also served as the reference genome for aligning sequencing data of 102 sperm from the boar. The sequencing data is 2.65Tb, yielding an average sequencing depth of 10.05X (ranging from 4.37X to 25.53X) (Supplementary Table 3). For the heterozygous sites, 99.76% of them were genotyped in the sperm population. The average coverage of heterozygous sites within the sperm was 49.49%, with a range spanning from 18.00% to 67.89% (Supplementary Table 4). Based on the sperm exhibiting the highest sequencing depth (S18–162, it covered 67.89% heterozygous sites), we constructed a bin map (Fig. 2a, analogous to Fig. 1b). This bin map enabled the detection of 19 false recombination sites (Fig. 2a). Upon the rectification of these false recombination sites, we produced the final bin map, comprised of 1,471 bins. These bins facilitated the phasing of heterozygous sites within each bin and subsequently served as haplotype makers for phased genome assembly (Fig. 2b, Supplementary Data 1).

### Phased genome assembly

**Fig. 3:**
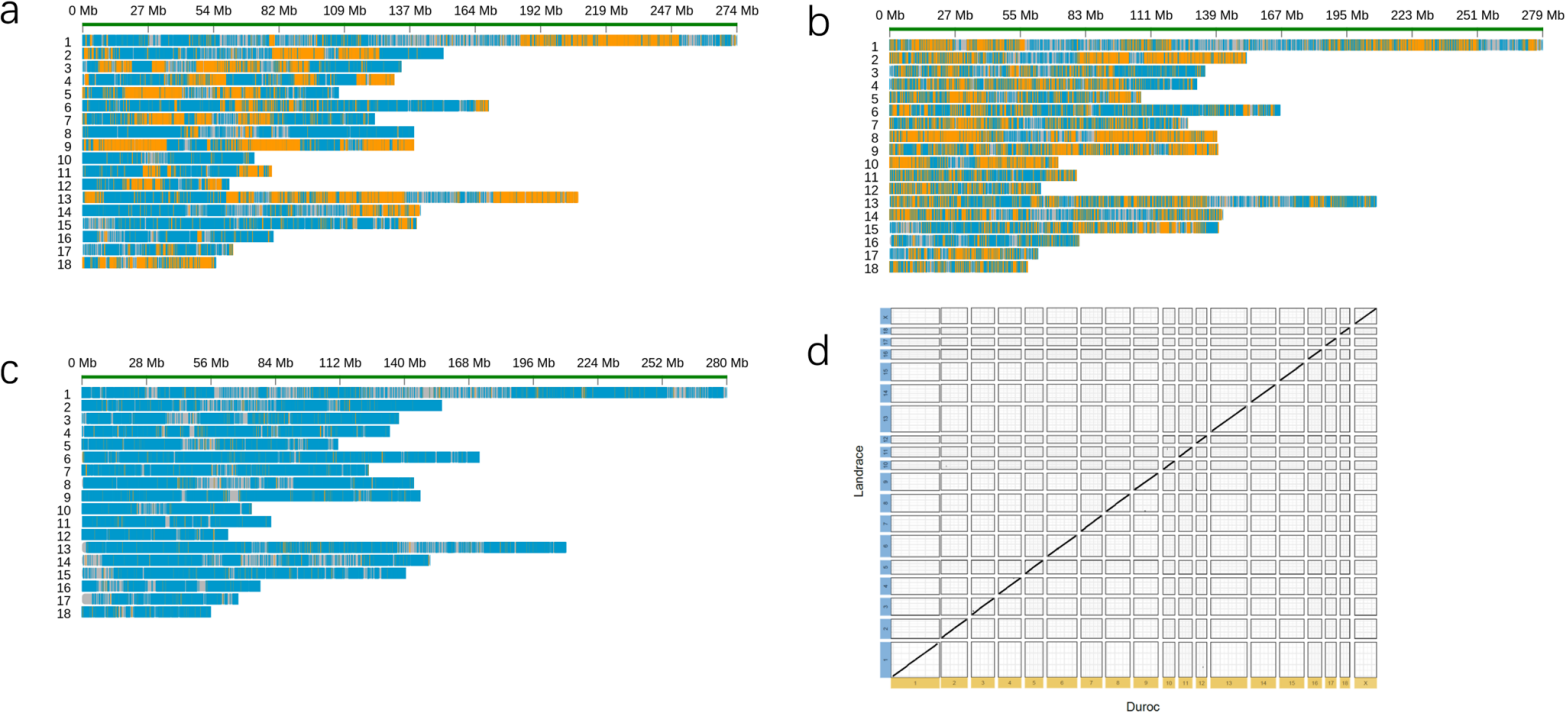
Comparison of different strategies of phased assembly. **a**–**c** The phased marker distribution in Dipasm assembly (**a**), Falcon phase assembly (**b**), and single sperm-based assembly (**c**). **d** Collinearity analysis of the single sperm assembly and the Duroc genome.

Using the phased markers, we categorized the PacBio HiFi reads into two groups, and the HiFi reads that were not classified were copied into two groups. The sequencing depths for these two groups are 25.42X and 25.26X, respectively (Supplementary Table 5). Employing five assembly software (Hifiasm, Next denovo, wtdbg2, flye, and Hicanu) on the classified HiFi reads, we observed that Hifiasm yielded the highest contig N50 and genome length (Supplementary Table 6). Consequently, we adopted Hifiasm for the final phased genome assembly. In addition, we compared our phased assembly strategy with other established pipelines like Dipasm and Falcon phase. All three strategies resulted in highly continuous and complete genomes (Table 1). While the contig N50 of the single-sperm pipeline was comparatively shorter, it yielded the longest assembly length (2.61 Gb, Table 1). However, upon assessing the output using phased markers, the results showed that Dipasm produced lengthy phased segments without achieving the chromosome level (Fig. 3a). Conversely, the Falcon phase failed to produce long-phased segments (Fig. 3b), echoing previous findings^1^. By leveraging the single-sperm sequencing method, we successfully obtained a chromosome-level phased genome (Fig. 3c), which exhibited strong collinearity with the Duroc genome (Fig. 3d). To evaluate the completeness of the phased genome, we conducted a Benchmarking Universal Single-Copy Orthologs (BUSCO) analysis against mammalian genes. The Landrace genome exhibited 96.4% completeness and 2% partial completeness among the 4104 vertebrate BUSCOs genes. By comparing the assembly of telomeres and centromeres between the genomes of Landrace and Duroc pigs, we found that the Landrace genome assembles more telomere sequences (Supplementary Fig. 3). This result represented the highest integrity achieved at the chromosomal level among available pig genomes to date (Supplementary Table 7), and the assembly outcomes conclusively demonstrated that the phased chromosomes derived from the single-sperm sequencing method were meticulously assembled, showcasing superior continuity and quality.

**Table 1:** The phased assembly of the Landrace pig

Feature	Single sperm		Falcon-phase		Dipasm	
	p0	p1	phased.0	phased.1	H1	H2
Number of scaffolds	532	242	465	464	3555	3659
Contig N50 (Mb)	16.58	17.40	29.56	29.65	25.46	24.64
Scaffold N50 (Mb)	139.47	139.87	139.74	139.89	139.09	139.12
Longest Scaffold (Mb)	277.04	285.09	279.69	279.78	275.92	275.89
Total scaffold length (Gb)	2.59	2.61	2.52	2.52	2.55	2.55
GC rate (%)	42.40	42.40	42.20	42.20	42.20	42.20
Illumina reads mapping rate (%)	99.61	99.82	99.79	99.79	99.80	99.79

### Genome annotation

Comparable to the Duroc genome, approximately 34.09% of the Landrace genome consisted of repeat contents (Supplementary Table 8). Public protein sequences from the genomes of six mammals, which include human, mouse, cattle, dog, goat, and Duroc pig, were employed as queries to search the Landrace genome for homology predictions. Then public data from nine tissues and one pooled RNA library from Landrace pig were used to optimize the gene annotation. Finally, we identified and predicted 21,982 protein-coding genes (PCGs), with functional annotations attributed to 97.82% of them (Supplementary Table 9). Our genomic analysis also revealed the presence of 725 ribosomal RNAs (rRNAs), 4496 transfer RNAs (tRNA), 852 microRNAs (miRNAs), and 1808 small nuclear RNAs (snRNAs) in the Landrace genome (Supplementary Table 10). Moreover, approximately 31.70% of the Landrace genome sequence was annotated as transposable elements (TEs) (Supplementary Table 11).

### Assisting assembly of Y chromosomes using Y chromosome-bearing sperm cells

The assembly of the Y chromosome in mammals has historically been challenging due to its highly repetitive sequence characteristics and the presence of homologous regions with the X chromosome^25^. However, leveraging the Y chromosome markers identified from the Y chromosome-bearing sperm cells (Supplementary Table 12), we were able to select the Y chromosome-specific PacBio HiFi reads. This approach allowed us to exclude the influence of X homologous regions, enabling the construction of a robust and accurate Y chromosome assembly. 66.33 Gb of Y chromosome-specific short reads were obtained for selecting Y-specific long reads. After aligning the 6.37 Gb candidate HiFi reads to the genome and removing the long reads aligned to autosomes, we finally obtained the Y chromosome sequence that spans 26.14 Mb in length and encompasses 57 intact PCG models. We successfully identified the presence of key genes such as ZFY and SRY. In humans, SRY and ZFY are linked to the Y chromosome^26^. Comparing the Landrace Y chromosome assembly with other published pig genomes at the chromosomal level, our assembly boasts the longest total length of non-N bases, indicative of its superior quality and completeness (Table 2).

**Table 2:** The comparison of the Y chromosome

Breed	Total Y chromosome length	Non-N bases length	N content	GC content	Gap number
Duroc (GCA_000003025.6)	43,547,828	15,568,313	64.25%	15.45%	415
Landrace	26,139,860	26,029,860	0.42%	42.69%	220
Bama (GCA_007644095.1)	4,685,480	4,573,506	2.39%	40.64%	30
Luchuan (CNP0001159)	14,970,572	12,084,736	19.28%	35.93%	84

The genome of the Luchuan pig was downloaded from the China National GenBank (CNGB; https://db.cngb.org/). The genomes of Duroc and Bama pigs were downloaded from the NCBI database.

### Genetic map construction

**Fig. 4:**
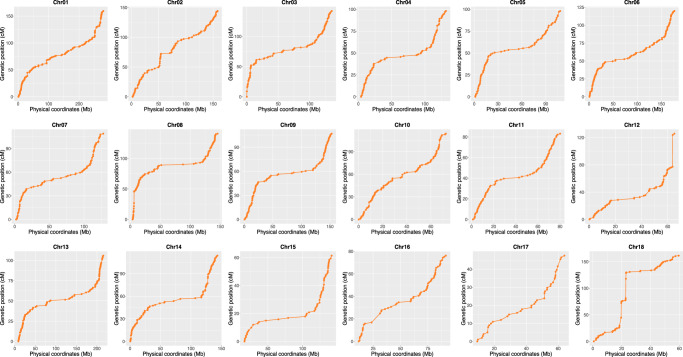
Comparison of genetic map and physical map. The X axes represents the physical location, and the Y axes represents the genetic distance.

For the first time, we have successfully constructed both a physical recombination map and a genetic map of pigs using the sperm population. This accomplishment holds significance for comprehending genetic recombination in pigs and facilitating the mapping of sperm-related phenotypes. Upon a comparative analysis of the recombination map and the genetic map, the results showed the recombination rate of the genome regions near the telomere is higher than regions near the centromere regions, aligning with prior research findings^27^ (Fig. 4). According to previous research, these phenomena reflect the cumulative evolutionary history of recombination^28^. Our study revealed that each sperm, on average, experienced 18 crossovers. This number contrasts with 12 in mice (inferred 2649 crossovers in 217 sperm)^29^ and 26 crossovers observed in human sperm^27^.

### Mapping of sperm motility traits

**Fig. 5:**
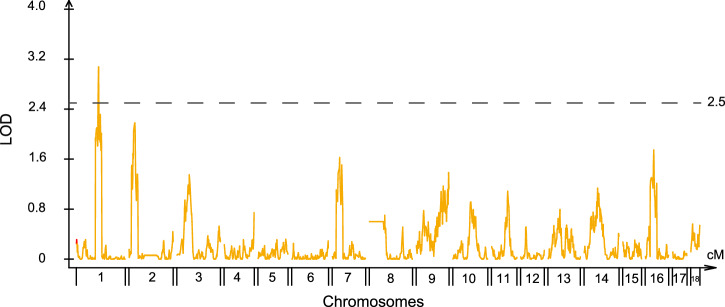
QTL mapping of sperm activity traits. The X axes represent the chromosome, and the Y axes represent the LOD values. The dashed line represents the significant threshold of LOD at 2.5. LOD is the log10 likelihood ratio comparing the hypothesis of a QTL at position versus that of no QTL.

Sperm motility is an essential characteristic that assesses the swimming ability of sperm. To measure sperm motility, a three-tiered grading system was conducted (See method). Utilizing the genetic map and 1471 bin markers (Supplementary Data 1 and 2), we successfully mapped the Quantitative Trait Loci (QTL) associated with sperm motility (Supplementary Table 13, Supplementary Data 3). The results showed that there is a peak with LOD of 3.08 at 107.40 Mb (genetic distance of 72.31 cM) in chromosome 1 (Fig. 5). The significant interval is approximately 5.30 Mb, with a range of 105.40–110.70 Mb (genetic distance of 71.31–73.31 cM) (Supplementary Data 3). However, considering the limited number of sperm samples and the precision of phenotype identification, the QTL results still need further validation. Nevertheless, our analysis validates the feasibility of employing single-sperm sequencing to construct a genetic map for studying sperm-related phenotypes. This approach provides opportunities to investigate the genetic foundations of sperm traits.

## Conclusion

In this study, combining Pacbio HiFi sequencing and single-sperm sequencing, a chromosome-level phased assembly of a male Landrace pig was generated. In the assembly process, we developed an approach for inferring parental haplotypes using single-sperm data, as well as assembling the Y chromosome by utilizing Y chromosome-bearing sperm. This enables us to understand the genetic contribution of parental haplotypes and achieve precise reconstruction of the Y chromosome sequence, which is important for understanding male-specific genetic traits and potential diseases. The construction of the pig genetic map and the QTL mapping of sperm motility trait demonstrate the potential of single-gamete sequencing in basic biological scientific research and sperm phenotype research.

## Methods

### Sample collection and PacBio sequencing

Fresh blood samples from a male Landrace pig (two years old) were collected for genome assembly. High-quality genomic DNA (gDNA) was extracted and assessed for purity and quantity using Nanodrop 1000 (Thermo Fisher) and Qubit (Thermo Fisher) assays. A library with an average insert size of ~15 kb was generated using the SMRTbell Express Template Prep Kit 2.0 (PacBio) and fractionated on the SageELF (Sage Science, Beverly, MA) into narrow library fractions. The library was then sequenced on 4 SMRT Cells 8 M on a Sequel II system (Pacbio) using 30 h movie times. Raw data were processed using the CCS algorithm (version 6.0.0, parameters: --minPasses 3 --all --max-length 50000) to generate highly accurate HiFi reads.

### Single sperm sequencing

Mature sperm cells were obtained from freshly ejaculated semen from the same Landrace boar, which was used for genome assembly. The sperms were diluted to ~1/mm^2 using PBS + 1%BSA on a petri dish. After phenotyping, every candidate single sperm was isolated by mouth pipetting and put into a reaction tube. The sperm were washed twice with PBS + 1%BSA before being lysed for 3 h in the lysis buffer, as described in a previous study^27^. The Multiple Annealing and Looping Based Amplification Cycles (MALBAC) technique was employed for single-cell whole genome amplification, with minor modifications^30^. For cell lysis, each individual cell was introduced into 5 μL of fresh cell lysis buffer, comprising 15 mM DTT, 10 mM Tris-EDTA, 1 mg/ml Qiagen protease, 0.3% Triton X-100, 200 mM KCL, and 0.25 μM GAT3G primer. The lysed cell underwent centrifugation for 1 min at 7000 rpm, followed by a 3 h incubation at 50 °C and protease inactivation at 70°C for 30 min.

Single-cell whole genome pre-amplification with Multiple Annealing and Looping Based Amplification Cycles (MALBAC). The MALBAC primer featured a common 27-nucleotide sequence: GTG AGT GAT GGT TGA GGT AGT GTG GAG. The process initiated with a pre-amplification, during which 30 μL of amplification mixture I (consisting of 1×Thermopol buffer, 0.1 mM dNTP, 133 mM Mg, 0.33 mM Primer1, and 0.33 mM Primer2) was added to PCR tubes containing the lysed single cell. This mixture was subjected to temperature cycles: 95 °C for 3.5 min, followed by 11 cycles of 4 °C for 50 s, 10 °C for 50 s, 20 °C for 50 s, 30 °C for 50 s, 40 °C for 45 s, 50 °C for 45 s, 65 °C for 4 min, 95 °C for 20 s, 58 °C for 20 s, and a 4 °C pause. The tubes were then rapidly cooled on ice.

In the exponential amplification phase, 30 μL of amplification mixture I (comprising 1×Thermopol buffer, 0.1 mM dNTP, 100 mM Mg, 0.67 μM primer, and 0.067 U/μl DeepVentR (exo-) polymerase) was introduced to PCR tubes with the pre-amplified samples from the previous step. This mixture underwent temperature cycles: 95 °C for 30 s, followed by 17 cycles of (95 °C for 20 s, 58 °C for 30 s, 72 °C for 3 min), then a final step of 72 °C for 5 min, 58 °C for 20 s, and a hold at 4 °C. This process facilitated the exponential amplification of DNA from the single sperm cell.

Following this procedure, the converted DNA was subjected to purification using Zymo-Spin columns (Zymo). Subsequently, the DNA was eluted in 50 μl of elution buffer. To ensure the quality and quantity of the DNA, assessment was performed using a Qubit High-Sensitivity dsDNA kit., the libraries were sequenced on the Illumina Novaseq sequencing platform.

### Sperm motility identification

To measure sperm motility, a three-tiered grading system was conducted. Grade 1 sperm: These sperm exhibited progressive motility, representing the highest level of motility. They demonstrated strong swimming abilities, moving swiftly in a straight trajectory. Grade 2 sperm (Non-Linear Motility): This category encompasses sperm that move forward, albeit in a curved or irregular manner. Their motion was not strictly linear. Grade 3 sperm: These sperm were characterized by non-progressive motility. Despite moving their tails, they did not make significant forward progress. Furthermore, within grade 3, there were immotile sperm that showed no movement whatsoever. All sperm were observed under a microscope at a magnification of 100X. Each sperm, after phenotyping, was mouth pipetted into a reaction tube and washed twice with PBS + 1%BSA before being lysed for 3 hours in the lysis buffer for subsequent genome sequencing.

### Hi-C library construction

Genomic DNA was extracted from the ear for the purpose of constructing the Hi-C library. The Hi-C fragment libraries were generated with insert sizes ranging from 300 to 700 base pairs (bp), and were subjected to sequence on the Illumina platform. The enzyme DpnII was employed to cleave at the recognition sequence “GATC”. Following sequencing, adapter sequences of the raw reads were trimmed, and paired-end reads with low quality were eliminated to yield clean reads using the fastp program (version 0.19.5)^31^ with default parameters.

### Initial genome assembly

To accomplish a diploid contig assembly of the Landrace genome. The initial genome assembly was generated using the Falcon assembler, followed by FALCON-Unzip^6^, integrated into the pbassembly tool suite (version 0.0.4). This resulted in a draft assembly consisting of primary contigs representing a partially phased haploid genome and haplotigs that represent phased alternative alleles for a subset of the genome. Two rounds of contig polishing were then performed. For the first round, as part of the FALCON-Unzip pipeline, primary contigs and secondary haplotigs were polished using haplotype-phased reads and the Quiver consensus caller. For the second round of polishing, the primary contigs and haplotigs were concatenated into a single reference and then mapped all raw reads to the combined assembly reference using pbmm2 (version 0.12.0), followed by consensus calling with Arrow (genomic consensus version 2.3.3). After the draft set of contigs was generated, a reference-guided scaffolding strategy was applied with RaGOO software^32^ based on the Sscrofa11.1 assembly. Finally, pilon (version 1.22)^33^ was used to correct errors introduced into the assembly from the long-read data.

### Sperm genotyping and phased assembly

The Illumina reads from the blood sample of the individual used for genome assembly were aligned to the unphased assembly by using the bwa mem algorithm (version 0.7.15-r1140)^34^. Then GATK (version 3.7-0-gcfedb67) software was used to detect the heterozygous sites of the whole genome^35^. To ensure the accuracy of heterozygous site identification, the filtering standard is as follows:

1. Quality value ≥ 30;
2. Sequencing depth of the minor allele ≥ 5;
3. *Chi*-square test was performed for the proportion of alleles, and the expected segregation ratio was 1:1, *P* = 0.05.

All sequencing data of single sperm were aligned to the partially phased Landrace genome by using bwa mem algorithm (version: 0.7.15-r1140)^34^ to get the alignment files. Then samtools mpileup (version 1.7)^36^ and Bcftools (version 0.1.19-96b5f2294a)^37^ were used to extract the corresponding alleles based on the heterozygous site information identified by the blood sample.

According to the quality control results, the sperm with the highest sequencing depth was used as a reference for the identification of recombination sites and haplotype inference. As shown in Fig. 1, assuming that sperm 1 is the reference sperm, other sperm can be compared with it to make a preliminary genotypic judgment based on whether the genotype is the same. Since sperm 1 may also have recombination sites, other sperm will present abnormal recombination at the recombination sites of sperm 1 (shown by the black line in Fig. 1) in the population; that is, all other sperm will recombine at this site. Based on this information, we can correct these false recombination sites and obtain complete chromosome haplotype information.

Using the sperm genotyping information, the phased markers and their flanking sequences were aligned to the HiFi reads. According to the haplotype information, HiFi reads were grouped. To obtain high-quality contigs, five approaches were used to test de novo genome assembly quality, including wtdbg2 (version 2.5)^38^, flye (version 2.8.3-b1695)^39^, HiCanu (version 2.1.1)^40^, Hifiasm (version 0.15.4_r343)^10^ and Nextdenovo (version v2.4.0) (https://github.com/Nextomics/NextDenovo). The parameters used for wtdbg2 were ‘-g 2500 m -x ccs -t 60 --edge-min 2 --rescue-low-cov-edges’. The parameters used for flye were ‘--genome-size 2.5 g --pacbio-corr --iterations 2’. The parameters used for HiCanu were ‘genomeSize=2.5 g useGrid=false maxThreads=60 -pacbio-hifi’. The default parameters were used for hifiasm assembly. Nextdenovo (version v2.4.0) was run with parameters of ‘read_type = hifi input_type = corrected genome_size = 2.5 g’. The assemblies yielded by hifiasm were used to remove heterozygous sequences by Purge haplotigs^41^ with the parameters -a 70. Subsequently, the Hi-C tech was employed to process contig assembly to obtain chromosome-level genome assembly. Detailed data processing procedures were provided as follows: (1) The paired-end Illumina reads were mapped onto the polished temporary genome assembly by using Hic-Pro (version 2.11.1)^42^ with default parameters to filter the raw Hi-C reads. Self-ligated, non-ligated, and other invalid reads (such as PCR amplification, random break, and extreme fragments) were discarded. (2) Juicer (version 1.6.2)^43^ and 3D-DNA (version 180114)^44^ were applied to cluster the genomic contig sequences into potential chromosomal groups. (3) JuiceBox (version 1.11.8)^45^ was employed to validate the contig orientation and to remove ambiguous fragments with the help of manual inspection.

### Genome completeness assessment

The completeness of the Landrace genome was assessed using the BUSCO program (version 5.0.2)^46^. The BUSCO analysis included 4104 mammalian genes with the “ -m genome” parameter. The telomere and centromeric repeats were identified by quarTeT^47^.

### Annotation of repeats

The interspersed repeats and low-complexity DNA sequences were identified using two methods, de novo repeat identification and known repeat searching against existing databases. RepeatModeler (version 1.0.8) was used to predict repeat sequences in the Landrace genome, RepeatMasker (version 4.0.7) (http://www.repeatmasker.org/) was then used to search the genome against the de novo transposable element (TE) library. The homology-based approach involved applying commonly used databases of known repetitive sequences, RepeatMasker (version 4.0.7) and the Repbase database^48^ were used to identify TE repeats in the assembled genome, and TEs were identified at both the DNA and protein levels, RepeatMasker was applied for DNA-level identification and RepeatProteinMasker was used to perform protein-level identification.

### Gene prediction and annotation

Protein sequences in the genome of six mammals, including human, mouse, cattle, dog, goat, and Duroc pig, were downloaded from the Ensembl database. Besides, the protein sequences of Luchuan pig were downloaded from the China National GenBank (CNGB; https://db.cngb.org/) under the accession of CNP0001159. Subsequently, these protein sequences were used as queries to search against the Landrace genome using GeMoMa (version 1.8)^49^. Homology predictions were denoted as “Homology-set”. To optimize the genome annotation, the raw reads of nine tissues and one pool RNA libraries from a Landrace pig (NCBI accession numbers: SRR3160015, SRR3160012, SRR3160008, SRR3160011, SRR3160014, SRR3160009, SRR3160017, SRR3160013, SRR3160010, and SRR3160016) were downloaded for further analyses. All raw reads were assessed using fastp (version 0.19.5)^31^. Then clean reads were mapped to the assembly using Hisat2 (version 2.0.1)^50^. The output bam files were merged using Samtools (version 1.10)^36^. Stringtie (version 1.2.2)^51^ and TransDecoder (version 3.0.1) (https://github.com/TransDecoder/TransDecoder) were employed to assemble the transcripts and identify candidate coding regions into gene models. Gene models created by RNA-seq were denoted as Stringtie -set. All gene models predicted were combined by EvidenceModeler (EVM)^52^ into a non-redundant set of gene structures. Finally, the produced gene models were refined with the Program to Assemble Spliced Alignment (PASA) (version 2.4.1)^53^. The integrated gene set was translated into amino-acid sequences. By using Diamond program (version 0.9.30.131)^54^ with an E-value cutoff of 1e-05, the amino-acid sequences were aligned to three public protein databases, SwissProt^55^, Kyoto Encyclopedia of Genes and Genomes (KEGG)^56^ and NCBI nonredundant database (NR). Moreover, BLAST^57^ was applied to search against Translation of European Molecular Biology Laboratory (Trembl) databases (E-value 1e-05). At last, we search protein domains through InterProScan (version 5.30)^58^ program. The Gene Ontology (GO) terms for each gene were extracted with InterProScan v5.30.

### Noncoding RNAs (ncRNAs) annotation

Four types of ncRNAs were annotated in the Landrace genome, including miRNA, tRNA, rRNA, and snRNA. The tRNA genes were predicted by tRNAscan-SE (version 1.3.1)^59^ with eukaryote parameters. The rRNA fragments were predicted by searching against vertebrate rRNA sequences using BLAST (version 2.2.24) with an E-value of 1e-5. The miRNA and snRNA genes were obtained by INFERNAL (version 1.1.1)^60^.

### Sperm-assisted Y chromosome assembly

First, 40 Y chromosome-bearing sperm cells were selected from the semen for high-throughput sequencing. By using the bwa mem algorithm (version 0.7.15-r1140)^34^, the clean reads of these 40 Y chromosome-bearing sperm cells were aligned to the Landrace genome. According to the alignment results, the Y chromosome-specific reads were obtained with samtools software (version 1.7) after removing the autosome alignment^36^. Next, the Y chromosome-specific reads were mapped to HiFi reads to obtain Y chromosome-specific long reads. The candidate HiFi long reads were mapped the Landrace genome again to remove the autosome alignments using minimap2^61^. Hifiasm (version 0.15.4_r343)^10^ was used to generate sex assembly from these reads using default parameters. After removing possible contaminants, redundant sequences were identified and removed using the Purge Haplotigs pipeline^41^, with the parameters -a 70. Last, we anchored the non-redundant sequences into scaffolds with Hi-C data. The protein-coding genes were predicted by miniport (https://github.com/lh3/miniprot) with genes from the Y chromosome of human, Duroc pig and goat genomes^62^.

### Genome alignment and collinearity analysis

Landrace pig assembly was aligned to the Duroc pig genome with MUMmer (v 3.23)^63^ using default parameters and the genomic alignment results were extracted with the delta-filter −1 -l 10000 parameters. R (v3.5.1) was used to visualize the collinear results.

### Genetic map construction and QTL mapping

Consecutive phased markers were used for recombination breakpoint detection with a sliding window approach^64^. The recombination map of each sperm was determined, and genotypes were determined for each 100-kb interval. Adjacent 100-kb intervals with the same genotype in each sperm were merged into a bin. The genetic map was constructed using 1481 recombination bins, and the genetic distance was calculated with the Kosambi mapping function^65^. Subsequently, QTL was identified using composite interval mapping (CIM) implemented in the Windows QTL Cartographer V2.5^66^ package. A 10-cM scan window was employed, and the walking speed was set as 1 cM. LOD values and R^2^ were determined based on likelihood ratio tests under a hypothesis allowing both additive and dominance effects. QTLs were identified based on LOD values equal to or higher than 2.5.

### Reporting summary

Further information on research design is available in the Nature Portfolio Reporting Summary linked to this article.

### Supplementary information

Supplementary information

Description of Additional Supplementary Files

Supplementary Data 1

Supplementary Data 2

Supplementary Data 3

Reporting summary

## Data Availability

The sequencing data for this project have been deposited in the NCBI Sequence Read Archive (SRA) (http://www.ncbi.nlm.nih.gov/sra) under accession number PRJNA977441. The genome sequences have been deposited into CNGB Sequence Archive (CNSA)^67^ of China National GeneBank DataBase (CNGBdb)^68^ with accession number CNP0004469. Gene annotation files were uploaded to Figshare (https://figshare.com/s/f37b58dfa53047f0b08d).

## Code availability

Codes used in this study were deposited to GitHub (https://github.com/Niuyongchao/sperm_phasing).
