# Supplementary material for: Haplotype-resolved assembly of a pig genome using single-sperm sequencing
Source: Commun Biol. 2024 Jun 18;7:738. doi: 10.1038/s42003-024-06397-x (PMC11189477; doi:10.1038/s42003-024-06397-x)
Supplement: Supplementary file 1 — Supplementary information [file 42003_2024_6397_MOESM1_ESM.pdf]

Supplementary Figures

Supplementary Figure 1. Schematic diagram of sperm haplotype production. Numbers 1-8 represent the chromosomes of No.1-8 sperm chromosomes, and the parental genotype was indicated by A, B and orange and blue colours, respectively.

Supplementary Figure 2. Heterozygote sites distribution along the autosomes.

Supplementary Figure 3. The distribution of the centromeres and telomeres in the Duroc and the Landrace genome.

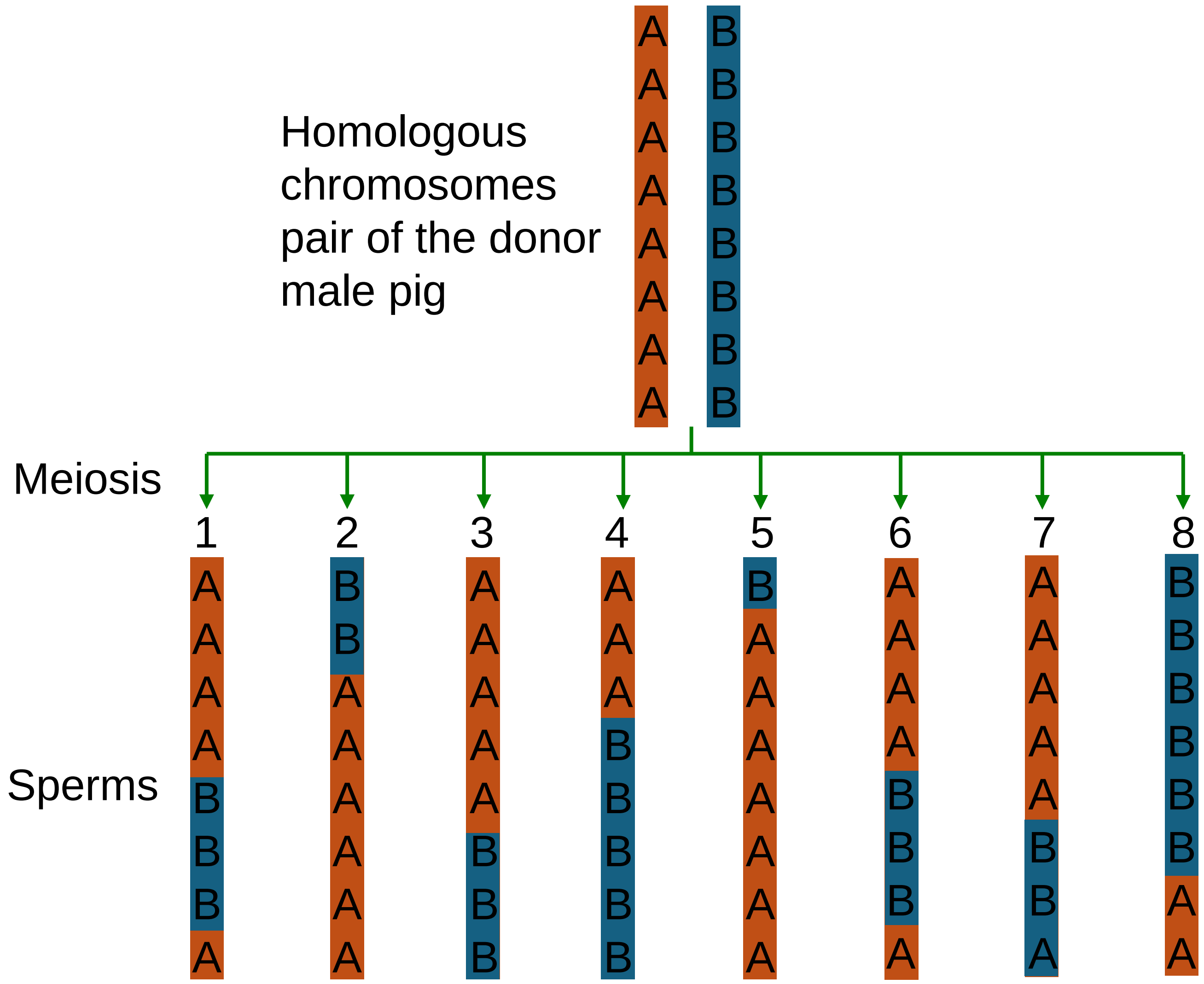

Supplementary Figure 1. Schematic diagram of sperm haplotype production. Numbers 1-8 represent the chromosomes of No.1-8 sperm chromosomes, and the parental genotype was indicated by A, B and orange and blue colours, respectively.

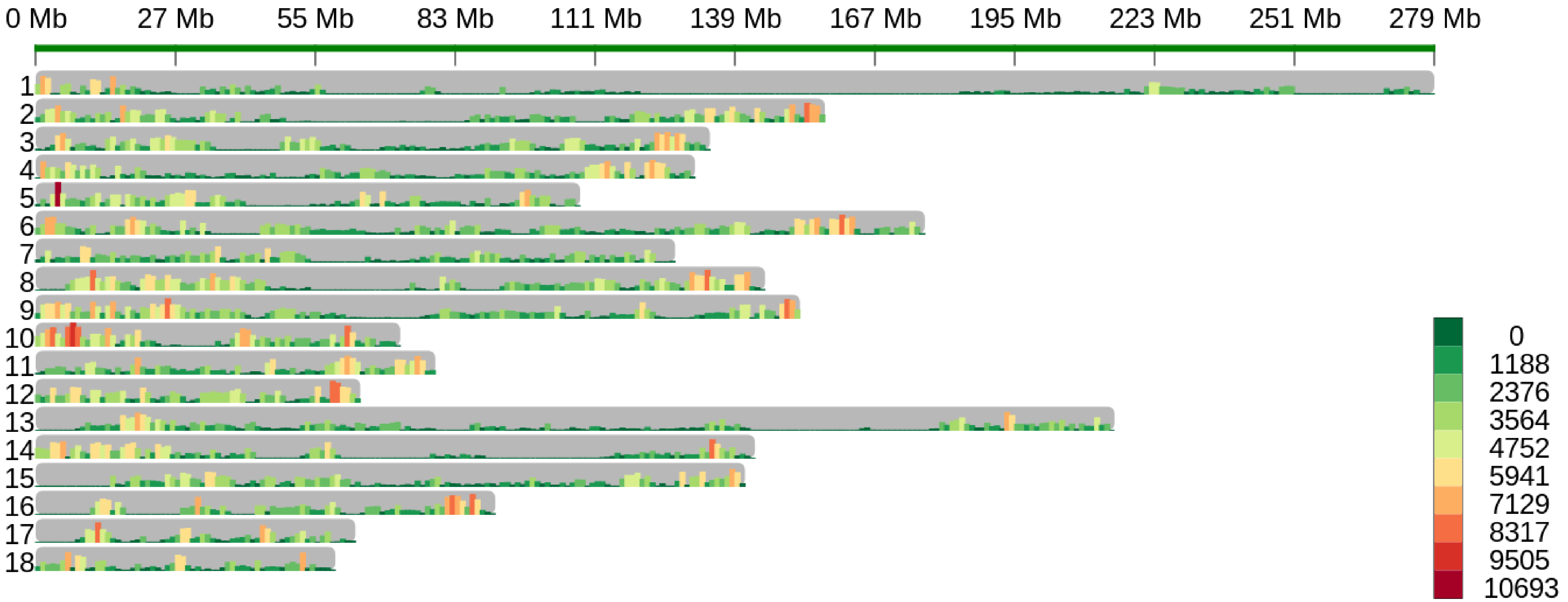

Supplementary Figure 2. Heterozygote sites distribution along the autosomes.

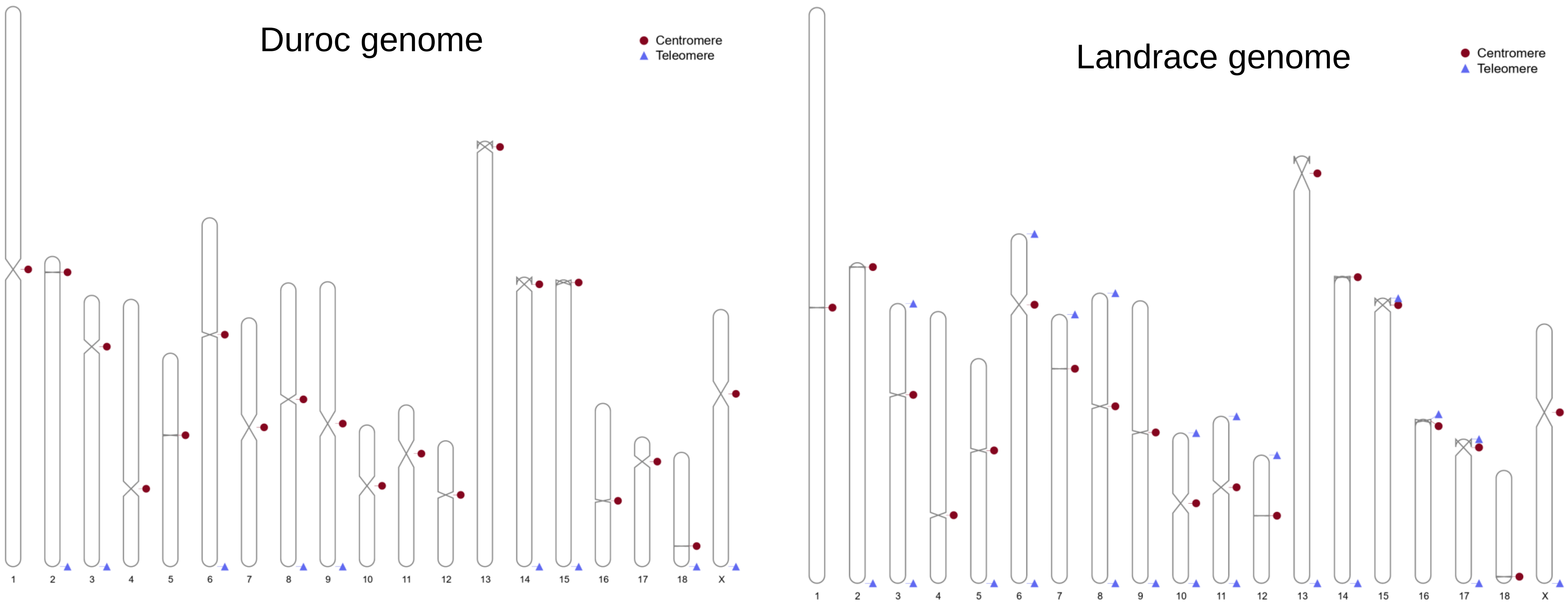

Supplementary Figure 3. The distribution of the centromeres and telomeres in the Duroc and the Landrace genome.

Supplementary Tables

Supplementary Table 1. Summary of sequencing data generated in this study for genome assembly.

Supplementary Table 2. Assembly features using Falcon unzip pipeline.

Supplementary Table 3. Summary of single sperm sequencing in this study.

Supplementary Table 4. Summary of single sperm heterozygous SNP coverage.

Supplementary Table 5. Statistic of the separated CCS reads.

Supplementary Table 6. The contig assembly quality of five methods based on p1 CCS reads.

Supplementary Table 7. Comparison of the four pig assemblies.

Supplementary Table 8. General statistics of repeats in the landrace and Duroc pig genome.

Supplementary Table 9. The number of Landrace pig genes with functional classification.

Supplementary Table 10. Non-coding RNAs in the Landrace pig assembly.

Supplementary Table 11. TEs content in the assembled Landrace pig genome.

Supplementary Table 12. The sequencing quality and read alignment statistic of 40 Y chromosome-bearing sperm cells.

Supplementary Table 13. The sperm motility of each sperm.

Supplementary Table 1. Summary of sequencing data generated in this study for genome assembly.

Libraries	Insert size	Raw data (Gb)	Clean data (Gb)	Read length (bp)	Sequence coverage (X)
Illumina reads	350 bp	271.05	237.79	150	95.12
PacBio reads	20 Kb	1,520.41	94.26	15,015	37.70
Hi-C	350bp	92.67	89.94	150	35.98

Note: The coverage was calculated using clean data and an estimated genome size of 2.5Gb.

Supplementary Table 2. Assembly features using Falcon unzip pipeline.

Assembly feature	Falcon unzip pipeline
Size of assembly	2.58Gb
Contig N50	29.52Mb
Contig number	1,438
GC content	42.40%
Complete BUSCOs	93.18%
NGS mapping Ratio	99.80%
NGS mapping coverage	99.96%

Supplementary Table 3. Summary of single sperm sequencing in this study.

Sample_ID	Reads(M)	Base(Gb)	GC(%)	Q20(%)	Sequencing_depth (X)*
17-111	75.36	11.30	43.48	97.60	4.37
17-133	76.80	11.52	42.77	97.18	4.46
17-80	77.69	11.65	43.05	97.64	4.51
14-57	79.80	11.97	41.09	98.02	4.63
18-191	89.14	13.37	43.40	97.77	5.17
18-200	101.26	15.19	42.29	97.71	5.88
60	110.97	16.65	40.79	97.49	6.44
67	121.80	18.27	41.07	97.63	7.07
B140	134.95	20.24	41.50	96.08	7.83
B143	140.76	21.11	40.85	96.14	8.17
A107	146.18	21.93	41.98	95.74	8.49
8B	147.68	22.15	41.05	97.72	8.57
D152	147.70	22.15	42.25	96.42	8.57
B176	148.82	22.32	43.27	95.99	8.64
50	149.14	22.37	40.24	97.56	8.66
A114	151.64	22.75	42.03	95.90	8.80
49B	152.05	22.81	41.22	97.45	8.83
46	152.23	22.83	40.70	97.42	8.84
286	152.62	22.89	43.35	97.54	8.86
23D	153.50	23.02	40.33	97.53	8.91
273	154.07	23.11	43.36	97.23	8.94
A129	154.69	23.20	43.37	96.19	8.98
S11A	154.99	23.25	40.73	97.75	9.00
298A	155.55	23.33	43.05	97.06	9.03
12B	155.57	23.34	40.75	97.09	9.03
24D	155.89	23.38	41.32	97.22	9.05
S4B	155.85	23.38	40.08	97.42	9.05
335D	156.41	23.46	42.28	97.65	9.08
237B	156.48	23.47	43.44	96.86	9.08
D177	156.51	23.48	42.72	96.63	9.09
262	157.40	23.61	41.65	97.69	9.14
59B	157.54	23.63	43.16	97.45	9.15
204B	158.31	23.75	43.23	97.32	9.19
87	158.58	23.79	43.08	97.26	9.21
88	159.65	23.95	42.50	97.56	9.27
291	161.67	24.25	43.75	97.74	9.39
82	161.99	24.30	44.19	97.53	9.40
296A	162.44	24.37	42.25	97.57	9.43
96	162.74	24.41	42.64	92.23	9.45
211D	163.11	24.47	43.42	97.33	9.47
33A	163.36	24.50	41.50	97.75	9.48
86	163.52	24.53	41.85	97.66	9.49
207D	163.63	24.55	43.39	97.42	9.50
B167	164.62	24.69	42.80	89.23	9.56
259	165.02	24.75	43.84	97.54	9.58
210A	165.58	24.84	43.23	97.38	9.61
136	167.56	25.13	41.66	97.49	9.73
25D	167.68	25.15	40.34	97.43	9.73
A115	168.09	25.21	42.97	96.51	9.76
318	168.34	25.25	43.21	97.49	9.77
222	168.87	25.33	43.28	97.70	9.80
131	169.01	25.35	41.44	97.59	9.81
94	169.05	25.36	42.11	97.18	9.81
316	169.12	25.37	42.46	97.68	9.82
294	169.55	25.43	41.81	97.62	9.84
B154	170.65	25.60	42.13	96.01	9.91
272	170.90	25.63	42.41	97.72	9.92
305D	171.25	25.69	41.90	95.35	9.94
324	171.89	25.78	43.05	97.55	9.98
134	172.45	25.87	42.51	97.44	10.01
47D	172.60	25.89	40.79	97.56	10.02

248	172.76	25.91	42.65	96.63	10.03
252	173.75	26.06	43.36	97.51	10.09
7B	177.57	26.64	40.93	97.54	10.31
43D	177.88	26.68	40.63	97.26	10.33
90	178.13	26.72	42.92	97.24	10.34
218	178.93	26.84	44.20	97.55	10.39
138	179.19	26.88	42.47	97.37	10.40
293	180.32	27.05	41.81	97.44	10.47
243	180.58	27.09	42.51	97.33	10.48
125	181.17	27.18	42.43	97.34	10.52
267	181.37	27.21	42.21	97.40	10.53
233D	181.66	27.25	43.95	97.44	10.55
247	182.45	27.37	42.92	97.59	10.59
85	184.28	27.64	42.84	97.19	10.70
271	185.11	27.77	42.56	97.52	10.75
S18B	187.31	28.10	40.54	97.34	10.88
B175	188.88	28.33	41.78	95.92	10.96
201A	188.93	28.34	42.80	97.24	10.97
303	189.94	28.49	42.81	97.42	11.03
D168	191.57	28.74	42.62	96.35	11.12
215B	191.87	28.78	42.77	97.13	11.14
135	192.49	28.87	42.56	97.37	11.17
137	194.13	29.12	42.38	96.61	11.27
A103	194.39	29.16	42.51	96.00	11.29
A110	194.93	29.24	42.62	95.91	11.32
117	195.15	29.27	43.61	96.31	11.33
315	197.64	29.65	43.09	97.39	11.47
B174	197.66	29.65	42.94	95.85	11.47
D155	198.62	29.79	41.87	96.60	11.53
S3	198.70	29.80	42.89	97.28	11.53
D163	200.13	30.02	44.32	96.59	11.62
239D	202.38	30.36	43.25	96.89	11.75
227	202.49	30.37	44.50	97.69	11.75
213A	206.58	30.99	43.17	97.51	11.99
246	206.60	30.99	42.22	97.67	11.99
230D	208.89	31.33	43.34	97.15	12.13
225B	224.99	33.75	43.53	97.13	13.06
B187	228.50	34.27	41.57	95.97	13.26
S14-19	384.90	54.65	40.43	97.53	21.15
S14-58	393.50	57.09	40.73	97.35	22.09
S18-162	457.67	65.97	42.91	97.39	25.53

*genome size was used as 2.58G

Supplementary Table 4. Summary of single sperm heterozygous SNP coverage.

Sample ID	SNP number(sperm, autosomes)	Hete SNP number(blood, autosomes)	Coverage (%)
117	3231227	5023492	64.32
125	3234362	5023492	64.38
7B	904351	5023492	18.00
49B	929474	5023492	18.50
A114	975060	5023492	19.41
43D	1089420	5023492	21.69
B140	1213396	5023492	24.15
B143	1281734	5023492	25.51
B175	1294134	5023492	25.76
B154	1307748	5023492	26.03
A107	1349205	5023492	26.86
B167	1360378	5023492	27.08
A115	1372222	5023492	27.32
D177	1373614	5023492	27.34
60	1378325	5023492	27.44
B176	1384327	5023492	27.56
D152	1394337	5023492	27.76
D168	1431321	5023492	28.49
B174	1464193	5023492	29.15
47D	1479875	5023492	29.46
A103	1482786	5023492	29.52
25D	1499723	5023492	29.85
B187	1502768	5023492	29.91
50	1512300	5023492	30.10
S18B	1514592	5023492	30.15
D155	1517903	5023492	30.22
S11A	1524959	5023492	30.36
D163	1528563	5023492	30.43
A110	1536409	5023492	30.58
S3	1651434	5023492	32.87
23D	1714787	5023492	34.14
12B	1770989	5023492	35.25
A129	1804368	5023492	35.92
8B	1845025	5023492	36.73
131	1897467	5023492	37.77
67	2098675	5023492	41.78
88	2166945	5023492	43.14
S4B	2282455	5023492	45.44
24D	2362834	5023492	47.04
33A	2392532	5023492	47.63
286	2436689	5023492	48.51
46	2454451	5023492	48.86
S14-19	2501849	5023492	49.80
59B	2680946	5023492	53.37
S14-58	2751349	5023492	54.77
227	2784534	5023492	55.43
17-133	2791403	5023492	55.57
252	2793776	5023492	55.61
87	2810355	5023492	55.94
14-57	2824481	5023492	56.23
94	2835634	5023492	56.45
86	2837581	5023492	56.49
222	2853221	5023492	56.80
233D	2855528	5023492	56.84

262	2892633	5023492	57.58
272	2900078	5023492	57.73
271	2903471	5023492	57.80
218	2933726	5023492	58.40
237B	2967987	5023492	59.08
273	2969404	5023492	59.11
259	2980463	5023492	59.33
267	2986793	5023492	59.46
296A	3005343	5023492	59.83
136	3014851	5023492	60.02
207D	3015120	5023492	60.02
294	3022407	5023492	60.17
17-111	3030654	5023492	60.33
316	3080126	5023492	61.31
318	3090392	5023492	61.52
210A	3094036	5023492	61.59
17-80	3107819	5023492	61.87
85	3113185	5023492	61.97
335D	3115099	5023492	62.01
246	3115569	5023492	62.02
215B	3118552	5023492	62.08
305D	3131223	5023492	62.33
293	3137644	5023492	62.46
298A	3140653	5023492	62.52
134	3150450	5023492	62.71
204B	3155740	5023492	62.82
96	3158908	5023492	62.88
82	3170513	5023492	63.11
137	3173538	5023492	63.17
291	3181266	5023492	63.33
138	3186822	5023492	63.44
90	3192159	5023492	63.54
211D	3196256	5023492	63.63
324	3200898	5023492	63.72
248	3207907	5023492	63.86
225B	3213662	5023492	63.97
18-200	3217266	5023492	64.04
239D	3218808	5023492	64.08
230D	3223336	5023492	64.17
243	3241183	5023492	64.52
303	3241359	5023492	64.52
18-191	3243832	5023492	64.57
213A	3262740	5023492	64.95
315	3269554	5023492	65.09
201A	3290942	5023492	65.51
247	3320293	5023492	66.10
135	3327964	5023492	66.25
S18-162	3410216	5023492	67.89

Supplementary Table 5. Statistic of the separated CCS reads.

Type	Total length	Total number	N50	Sequencing depth (X)
p0	63,551,270,909	4,223,216	14,876	25.42
p1	63,160,451,496	4,128,843	15,007	25.26

Note: The depth was calculated using an estimated genome size of 2.5Gb.

Supplementary Table 6. The contig assembly quality of five methods based on p1 CCS reads.

Assembly feature	hifiasm	Next-denovo	wtdbg2	flye	HiCanu
Number of contigs	892	1,454	1,569	2,925	39,567
Contig N50	15,935,305	15,399,018	13,006,558	6,713,478	3,428,592
Longest contig	67,391,919	55,240,012	43,891,956	25,908,380	37,513,101
Total contigs length	2,653,017,232	2,554,376,865	2,403,156,629	2,487,141,329	3,777,757,263
GC rate	42.50%	42.20%	41.90%	42.20%	42.70%

Note: The statistics here are the original output results of the software without removing redundant sequences and processing HiC splicing

Supplementary Table 7. Comparison of the four pig assemblies.

**Overview statics**	Landrace (p1)	Duroc (GCA_000003025.6)	Luchuan (CNP0001159)	Bama (GCA_007644095.1)
Length of genome (bp)	2,613,091,380	2,501,912,388	2,582,939,082	2,491,053,062
Length of chromosome (bp)	2,479,818,230	2,435,262,063	2,482,118,608	2,436,957,124
Contig N50 length (bp)	17,401,331	41,891,233	18,025,403	1,009,524
Scaffold N50 length (bp)	139,868,373	138,966,237	140,090,008	140,438,739
**BUSCO assessment**				
C (%)	96.40	96.00	95.10	93.90
F (%)	2.00	2.40	2.90	3.20
M (%)	1.60	1.60	2.00	2.90

Note: The chromosome-level of Changbai pig genome was generated by Hi-C data. Luchuan pig genome was downloaded from the China National GenBank (CNGB; https://db.cngb.org/), and other genomes were from NCBI database. BUSCOs analysis included 4,104 BUSCO mammalian genes, BUSCO were run with the " -m genome" parameters. C: Complete BUSCOs;F: Fragmented BUSCOs; M: Missing BUSCOs

Supplementary Table 8. General statistics of repeats in the landrace and Duroc pig genome.

Method	Landrace (p1)		Duroc	
	Repeat Size	% of genome	Repeat Size	% of genome
Repeatmasker	531,936,660	20.36	516,352,486	20.64
Proteinmask	249,982,056	9.57	245,722,633	9.82
De novo	804,089,955	30.77	790,087,463	31.58
**Total**	890,793,865	34.09	863,051,487	34.50

Supplementary Table 9. The number of Landrace pig genes with functional classification.

Database	Number annotated	Percentage annotated
NR	21,826	97.13%
Swiss-Prot	21,568	95.98%
KEGG	19,146	85.20%
TrEMBL	21,898	97.45%
Interpro	21,585	96.06%
Total	21,982	97.82%

Supplementary Table 10. Non-coding RNAs in the Landrace pig assembly.

Type		Number	Average length(bp)	Total length(bp)	% of genome
miRNA		852	79	67,507	0.002584
tRNA		4,496	76	339,887	0.013008
rRNA	rRNA	725	274	198,304	0.007589
	18S	69	925	63,856	0.002444
	28S	358	311	111,399	0.004263
	5.8S	31	144	4,478	0.000171
	5S	267	70	18,571	0.000711
snRNA	snRNA	1,808	111	201,240	0.007702
	CD-box	473	92	43,506	0.001665
	HACA-box	239	136	32,524	0.001245
	splicing	1,075	113	121,346	0.004644

Note: Four classes of ncRNAs, namely microRNA (miRNA), transfer RNA (tRNA), ribosomal RNA (rRNA) and small nuclear RNA (snRNA), were identified.The rRNAs were divided into four sub-classes based on their molecular weight (18S, 28S, 5.8S and 5S), whereas the snRNAs contain the sub-classes of CD-box, HACA-box and spliceosomal RNA (splicing).

Supplementary Table 11. TEs content in the assembled Landrace pig genome.

Type	Repbase TEs		TE protiens		*De novo*		Combined TEs*	
	Length (bp)	% in genome	Length (bp)	% in genome	Length (bp)	% in genome	Length (bp)	% in genome
**DNA**	21,661,731	0.83	4,589,230	0.18	33,756,893	1.29	43,283,571	1.66
**LINE**	435,267,878	16.66	236,402,733	9.05	571,274,571	21.86	640,227,037	24.50
**SINE**	6,286,814	0.24	0	0.00	22,655,691	0.87	24,483,538	0.94
**LTR**	73,282,319	2.80	9,029,220	0.35	103,621,256	3.97	130,437,938	4.99
**†Unknown**	-	0.00	-	0.00	18,642,282	0.71	18,642,282	0.71
**Total**	531,936,660	20.36	249,982,056	9.57	741,629,665	28.38	828,333,575	31.70

Note: This statistical table does not contain Tandem Repeats, some elements may partly include another element domain.
*Combined: the non-redundant consensus of all repeat prediction/classification methods employed.
†Unknown: the predicted repeats that cannot be classified by RepeatMasker;
LINE, long interspersed nuclear elements; SINE, short interspersed nuclear elements; LTR, long terminal repeat.

Supplementary Table 12. The sequencing quality and read alignment statistic of 40 Y chromosome-bearing sperm cells.

Sample	Total reads	Total Length	%GC	%Q20	%Q30	Genome mapping rate	X chromosome depth	SRY depth
125	181,170,948	27,175,642,200	42.43	97.34	92.91	97.49%	0.0258	176
131	169,011,798	25,351,769,700	41.44	97.59	93.48	92.62%	0.031	61
134	172,448,428	25,867,264,200	42.51	97.45	93.24	97.25%	0.0251	29
135	192,490,246	28,873,536,900	42.56	97.37	93.11	97.64%	0.0224	31
14-57	79,798,794	11,969,819,100	41.09	98.02	94.50	97.21%	0.0252	72
17-111	75,355,012	11,303,251,800	43.48	97.60	93.66	97.50%	0.0253	25
17-133	76,796,414	11,519,462,100	42.77	97.18	92.69	94.55%	0.0274	77
18-191	89,137,202	13,370,580,300	43.40	97.78	93.91	97.76%	0.0264	41
204B	158,309,332	23,746,399,800	43.23	97.32	93.02	97.38%	0.024	81
207D	163,633,546	24,545,031,900	43.39	97.42	93.23	97.00%	0.0274	180
211D	163,113,664	24,467,049,600	43.42	97.33	93.12	97.92%	0.0249	233
213A	206,578,794	30,986,819,100	43.16	97.51	93.19	97.04%	0.028	235
215B	191,865,480	28,779,822,000	42.77	97.13	92.62	97.81%	0.0266	79
218	178,927,398	26,839,109,700	44.20	97.55	93.52	97.49%	0.0259	208
225B	224,991,516	33,748,727,400	43.53	97.13	92.60	97.05%	0.0266	110
230D	208,891,474	31,333,721,100	43.33	97.15	92.56	98.02%	0.0248	56
237B	156,475,034	23,471,255,100	43.45	96.86	91.53	93.83%	0.0274	170
239D	202,380,194	30,357,029,100	43.25	96.89	92.12	97.60%	0.0282	209
23D	153,499,734	23,024,960,100	40.33	97.53	93.17	97.57%	0.0245	42
246	206,598,772	30,989,815,800	42.23	97.67	93.69	98.21%	0.0237	261
247	182,453,564	27,368,034,600	42.92	97.59	93.51	97.99%	0.0256	43
252	173,754,026	26,063,103,900	43.37	97.51	93.23	91.67%	0.0261	221
259	165,023,314	24,753,497,100	43.84	97.55	93.52	97.25%	0.0249	14
272	170,896,512	25,634,476,800	42.41	97.72	93.84	98.15%	0.0255	115
273	154,074,572	23,111,185,800	43.36	97.23	92.77	98.14%	0.0268	77
286	152,618,984	22,892,847,600	43.35	97.53	93.44	96.75%	0.0271	41
293	180,318,758	27,047,813,700	41.81	97.44	93.12	97.18%	0.0232	102
296A	162,439,616	24,365,942,400	42.25	97.58	93.47	97.31%	0.0254	85
298A	155,546,142	23,331,921,300	43.05	97.05	92.38	97.21%	0.0239	92
335D	156,413,890	23,462,083,500	42.27	97.65	93.28	97.59%	0.0266	51
33A	163,357,914	24,503,687,100	41.50	97.75	93.94	98.27%	0.026	98
46	152,226,866	22,834,029,900	40.70	97.42	93.11	98.05%	0.0265	44
47D	172,603,072	25,890,460,800	40.79	97.56	93.39	97.84%	0.0239	32
59B	157,536,216	23,630,432,400	43.16	97.45	93.22	97.99%	0.026	121
7B	177,569,722	26,635,458,300	40.93	97.55	93.53	93.91%	0.0269	10
87	158,576,200	23,786,430,000	43.08	97.27	92.86	96.64%	0.0271	107
88	159,645,410	23,946,811,500	42.50	97.56	93.54	93.84%	0.0287	152
96	162,738,890	24,410,833,500	42.64	92.22	81.87	97.07%	0.0262	85
S11A	154,988,154	23,248,223,100	40.73	97.75	93.91	96.96%	0.0256	44
S3	198,696,526	29,804,478,900	42.89	97.28	92.89	95.28%	0.0226	188

Supplementary Table 13. The sperm motility of each sperm.

Sample_ID	Sperm motility levels
117	3
125	2
12B	2
131	3
134	2
135	3
136	2
137	3
138	2
14-57	-
17-111	-
17-133	-
17-80	-
18-191	-
18-200	-
201A	1
204B	2
207D	3
210A	1
211D	3
213A	1
215B	2
218	1
222	1
225B	2
227	1
230D	3
233D	3
237B	2
239D	3
23D	3
243	1
246	3
247	1
248	1
24D	3
252	1
259	3
25D	3
262	3
267	3
271	2
272	2
273	2
286	3
291	3
293	3
294	3
296A	1
298A	1
303	3

305D	3
315	2
316	2
318	2
324	2
335D	3
33A	1
43D	3
46	1
47D	3
49B	2
50	1
59B	2
60	3
67	3
7B	2
82	2
85	2
86	1
87	1
88	1
8B	2
90	2
94	1
96	1
A103	1
A107	1
A110	1
A114	1
A115	1
A129	1
B140	2
B143	2
B154	2
B167	2
B174	2
B175	2
B176	2
B187	2
D152	3
D155	3
D163	3
D168	3
D177	3
S11A	1
S14-19	-
S14-58	-
S18-162	-
S18B	2
S3	3
S4B	2

Note:
1. Grade 1 sperm.
2. Grade 2 sperm.
3. Grade 3 sperm.
"-". Missing data.
