# Supplementary material for: Haplotype-resolved assembly of a pig genome using single-sperm sequencing
Source: Commun Biol. 2024 Jun 18;7:738. doi: 10.1038/s42003-024-06397-x (PMC11189477; doi:10.1038/s42003-024-06397-x)
Supplement: Supplementary file 2 — Description of Additional Supplementary Files [file 42003_2024_6397_MOESM2_ESM.pdf]

Description of Additional Supplementary Files

File name: Supplementary Data 1

Description: The bin markers of the sperm population.

File name: Supplementary Data 2.

Description: Genetic map of the sperm population.

File name: Supplementary Data 3.

Description: The QTL results of sperm motility.
