# Supplementary material for: Haplotype-resolved assembly of a pig genome using single-sperm sequencing
Source: Commun Biol. 2024 Jun 18;7:738. doi: 10.1038/s42003-024-06397-x (PMC11189477; doi:10.1038/s42003-024-06397-x)
Supplement: Supplementary file 6 — Reporting summary [file 42003_2024_6397_MOESM6_ESM.pdf]

Corresponding author(s): Zhonglin Tang
Last updated by author(s): 2024-4-30

Reporting Summary

Nature Portfolio wishes to improve the reproducibility of the work that we publish. This form provides structure for consistency and transparency in reporting. For further information on Nature Portfolio policies, see our Editorial Policies and the Editorial Policy Checklist.

Statistics

For all statistical analyses, confirm that the following items are present in the figure legend, table legend, main text, or Methods section.

n/a	Confirmed	
☒	☐	The exact sample size (*n*) for each experimental group/condition, given as a discrete number and unit of measurement
☒	☐	A statement on whether measurements were taken from distinct samples or whether the same sample was measured repeatedly
☐	☒	The statistical test(s) used AND whether they are one- or two-sided<br>*Only common tests should be described solely by name; describe more complex techniques in the Methods section.*
☒	☐	A description of all covariates tested
☒	☐	A description of any assumptions or corrections, such as tests of normality and adjustment for multiple comparisons
☒	☐	A full description of the statistical parameters including central tendency (e.g. means) or other basic estimates (e.g. regression coefficient) AND variation (e.g. standard deviation) or associated estimates of uncertainty (e.g. confidence intervals)
☐	☒	For null hypothesis testing, the test statistic (e.g. F, t, r) with confidence intervals, effect sizes, degrees of freedom and P value noted<br>*Give P values as exact values whenever suitable.*
☒	☐	For Bayesian analysis, information on the choice of priors and Markov chain Monte Carlo settings
☒	☐	For hierarchical and complex designs, identification of the appropriate level for tests and full reporting of outcomes
☒	☐	Estimates of effect sizes (e.g. Cohen's *d*, Pearson's *r*), indicating how they were calculated

Our web collection on statistics for biologists contains articles on many of the points above.

Software and code

Policy information about availability of computer code

Data collection

Pacbio Raw data were processed using the CCS algorithm (version 6.0.0, parameters: --minPasses 3 --all --max-length 50000) to generate highly accurate HiFi reads.

Data analysis

The initial genome assembly was generated using the Falcon assembler, followed by FALCON-Unzip, integrated into the pbassembly tool suite (version 0.0.4). Primary contigs and secondary haplotigs were polished using haplotype-phased reads and the Quiver consensus caller. pbmm2 (version 0.12.0), followed by consensus calling with Arrow (genomic consensus version 2.3.3) were used for the second round of polishing. After the draft set of contigs was generated, a reference-guided scaffolding strategy was applied with RaGOO software based on the Sscrofa11.1 assembly. Finally, pilon (version 1.22) was used to correct errors introduced into the assembly from the long read data.
The Illumina Reads from the blood sample of the individual used for genome assembly were aligned to the unphased Landrace assembly by using the bwa mem algorithm (version 0.7.15-r1140). Then GATK (version 3.7-0-gcfedb67) software was used to detect the heterozygous sites of the whole genome. All the sequencing data of single sperm were aligned to the partially phased Landrace genome by using bwa mem algorithm (version: 0.7.15-r1140) to get the alignment files. Then samtools mpileup (version 1.7) and Bcftools (version 0.1.19-96b5f2294a) were used to extract the corresponding alleles based on the heterozygous site information identified by the blood sample. To obtain high-quality contigs, 5 approaches were used to test de novo genome assembly quality, including wtdbg2 (version 2.5), flye (version 2.8.3-b1695), HiCanu (version 2.1.1), Hifiasm (version 0.15.4_r343) and Nextdenovo (version v2.4.0) (https://github.com/Nextomics/NextDenovo). the Hi-C tech was employed to process contig assembly to obtain chromosome-level genome assembly using Hic-Pro (version 2.11.1), Juicer (version 1.6.2), 3D-DNA (version 180114) and JuiceBox (version 1.11.8). The completeness of the Landrace genome was assessed using the Benchmarking Universal Single-Copy Ortholog (BUSCO) program (version.5.0.2). The telomere and centromeric repeats were identified by quarTeT. RepeatModeler (version 1.0.8) was used to predict repeat sequences in the landrace genome, RepeatMasker (version 4.0.7) (http://www.repeatmasker.org/) was then used to search the genome against the de novo transposable element (TE) library. RepeatProteinMasker was used to perform protein-level identification. Protein sequences were used as queries to search against the Landrace genome using

GeMoMa (version 1.8). RNA reads were mapped to the assembly using Hisat2 (version 2.0.1)51. The output bam files were merged using Samtools (version 1.10). Stringtie (version 1.2.2) and TransDecoder (version 3.0.1) (https://github.com/TransDecoder/TransDecoder) were employed to assemble the transcripts and identify candidate coding regions into gene models. All gene models predicted were combined by EvidenceModeler (EVM). the produced gene models were refined with the Program to Assemble Spliced Alignment (PASA) (version 2.4.1). By using Diamond program (version 0.9.30.131) the amino-acid sequences were aligned to the public protein databases. BLAST was applied to search against Translation of European Molecular Biology Laboratory (Trembl) databases (E-value 1e-05). We search protein domains through InterProScan (version 5.30) program. The Gene Ontology (GO) terms for each gene were extracted with InterProScan v5.30. The tRNA genes were predicted by tRNAscan-SE (version 1.3.1) with eukaryote parameters. The rRNA fragments were predicted by searching against vertebrate rRNA sequences using BLAST (version 2.2.24) with an E-value of 1e-5. The miRNA and snRNA genes were obtained by INFERNAL (version 1.1.1).
For the Y chromosome assembly, bwa mem algorithm (version 0.7.15-r1140) was used for reads alignment. The Y chromosome-specific reads were obtained with samtools software (version 1.7) after removed the autosome alignment. The candidate HiFi long reads were mapped the Landrace genome again to remove the autosome alignments using minimap2. Hifiasm (version 0.15.4_r343) was used to generate sex assembly from these reads using default parameters. After removing possible contaminants, redundant sequences were identified and removed using the Purge Haplotigs pipeline, with the parameters -a 70. The protein-coding genes were predicted by miniport (https://github.com/lh3/miniprot) with genes from the Y chromosome of human, Duroc pig and goat genomes.
MUMmer (v 3.23) and R (v3.5.1) was used to genome alignment and visualization.
The genetic map was constructed using 1,481 recombination bins, and the genetic distance was calculated with the Kosambi mapping function. QTL was identified using composite interval mapping (CIM) implemented in the Windows QTL Cartographer V2.567 package.
The custom codes used in this study were deposited to github (https://github.com/Niuyongchao/sperm_phasing).

For manuscripts utilizing custom algorithms or software that are central to the research but not yet described in published literature, software must be made available to editors and reviewers. We strongly encourage code deposition in a community repository (e.g. GitHub). See the Nature Portfolio guidelines for submitting code & software for further information.

Data

Policy information about availability of data

All manuscripts must include a data availability statement. This statement should provide the following information, where applicable:

- Accession codes, unique identifiers, or web links for publicly available datasets
- A description of any restrictions on data availability
- For clinical datasets or third party data, please ensure that the statement adheres to our policy

The sequencing data for this project have been deposited in the NCBI Sequence Read Archive (SRA) (http://www.ncbi.nlm.nih.gov/sra) under accession number PRJNA977441. The genome sequences have been deposited into CNGB Sequence Archive (CNSA) of China National GeneBank DataBase (CNGBdb) with accession number CNP0004469. Gene annotation files were uploaded to Figshare (https://figshare.com/s/f37b58dfa53047f0b08d).

Human research participants

Policy information about studies involving human research participants and Sex and Gender in Research.

Reporting on sex and gender	*Use the terms sex (biological attribute) and gender (shaped by social and cultural circumstances) carefully in order to avoid confusing both terms. Indicate if findings apply to only one sex or gender; describe whether sex and gender were considered in study design whether sex and/or gender was determined based on self-reporting or assigned and methods used. Provide in the source data disaggregated sex and gender data where this information has been collected, and consent has been obtained for sharing of individual-level data; provide overall numbers in this Reporting Summary. Please state if this information has not been collected. Report sex- and gender-based analyses where performed, justify reasons for lack of sex- and gender-based analysis.*
Population characteristics	*Describe the covariate-relevant population characteristics of the human research participants (e.g. age, genotypic information, past and current diagnosis and treatment categories). If you filled out the behavioural & social sciences study design questions and have nothing to add here, write "See above."*
Recruitment	*Describe how participants were recruited. Outline any potential self-selection bias or other biases that may be present and how these are likely to impact results.*
Ethics oversight	*Identify the organization(s) that approved the study protocol.*

Note that full information on the approval of the study protocol must also be provided in the manuscript.

Field-specific reporting

Please select the one below that is the best fit for your research. If you are not sure, read the appropriate sections before making your selection.

☒ Life sciences ☐ Behavioural & social sciences ☐ Ecological, evolutionary & environmental sciences

For a reference copy of the document with all sections, see nature.com/documents/nr-reporting-summary-flat.pdf

Life sciences study design

All studies must disclose on these points even when the disclosure is negative.

Sample size	102 sperm were collected for sequencing, genotyping, phenotyping and QTL analysis.
Data exclusions	No data were excluded.
Replication	NA
Randomization	Sperm were selected randomly.
Blinding	NA

Reporting for specific materials, systems and methods

We require information from authors about some types of materials, experimental systems and methods used in many studies. Here, indicate whether each material, system or method listed is relevant to your study. If you are not sure if a list item applies to your research, read the appropriate section before selecting a response.

Materials & experimental systems

n/a	Involved in the study	
☒	☐	Antibodies
☒	☐	Eukaryotic cell lines
☒	☐	Palaeontology and archaeology
☐	☒	Animals and other organisms
☒	☐	Clinical data
☒	☐	Dual use research of concern

Methods

n/a	Involved in the study	
☒	☐	ChIP-seq
☒	☐	Flow cytometry
☒	☐	MRI-based neuroimaging

Animals and other research organisms

Policy information about studies involving animals; ARRIVE guidelines recommended for reporting animal research, and Sex and Gender in Research

Laboratory animals	A 2-year-old male Landrace pig was used in this study.
Wild animals	The study did not involve wild animals.
Reporting on sex	Male
Field-collected samples	NA
Ethics oversight	The animal experiments in this study were approved by the Institutional Animal Care and Use Committee of Agricultural Genomics Institute at Shenzhen, Chinese Academy of Agricultural Sciences (AGIS-ER-2023-003). We have complied with all relevant ethical regulations for animal use.

Note that full information on the approval of the study protocol must also be provided in the manuscript.
